# Spontaneous Resolution of Congenital Dural Venous Sinus Ectasia Associated With Polymicrogyria—Case Report

**DOI:** 10.3389/fped.2022.822551

**Published:** 2022-02-28

**Authors:** Zuzanna Kozłowska, Paulina Komasińska, Barbara Steinborn, Kinga Toboła-Wróbel, Marek Pietryga, Marta Szymankiewicz-Breborowicz, Tomasz Szczapa, Monika Bekiesińska-Figatowska

**Affiliations:** ^1^Department of Neonatology, Neonatal Biophysical Monitoring and Cardiopulmonary Therapies Research Unit, Poznan University of Medical Sciences, Poznan, Poland; ^2^Department of Developmental Neurology, Poznan University of Medical Sciences, Poznan, Poland; ^3^Department of Obstetrics and Women's Health, Poznan University of Medical Sciences, Poznan, Poland; ^4^Department of Newborns' Infectious Diseases, Chair of Neonatology, Poznan University of Medical Sciences, Poznan, Poland; ^5^Department of Diagnostic Imaging, Institute of Mother and Child, Warsaw, Poland

**Keywords:** neonate, neuroimaging, cortical malformations, torcular herophili, central nervous system

## Abstract

Dural venous sinus ectasia belongs to a rare group of venous sinus malformations of unknown origin and uncertain prognosis. We report the first patient with idiopathic congenital ectasia of the confluence of sinuses with thrombosis associated with bilateral polymicrogyria. It may highlight the causative relation between ischemia within the central nervous system due to torcular herophili ectasia with thrombosis in early pregnancy and the development of cortical malformations in neonates. We also highlight the role of MR neuroimaging in the diagnosis of these entities.

## Introduction

Dural venous sinus ectasia is a rare vascular defect representing a group of cerebral venous sinus malformations. It is mostly detected prenatally and located in the vicinity of the confluence of sinuses, an area also referred to as torcular herophili (1). The lesion results from venous sinus widening of an unknown origin. It may be associated with meningeal arteriovenous fistula, generalized ischemia, increased intracranial pressure leading to compression of the central nervous system (CNS), intracranial bleeding, consumptive coagulopathy, and circulatory failure (1). Prognosis for survival and psychomotor development is uncertain (1–3). We present the first case report of a patient with congenital ectasia of the confluence of sinuses with thrombosis diagnosed prenatally, which might have led to bilateral polymicrogyria (PMG) development.

## Case Presentation

**Figure 1:**
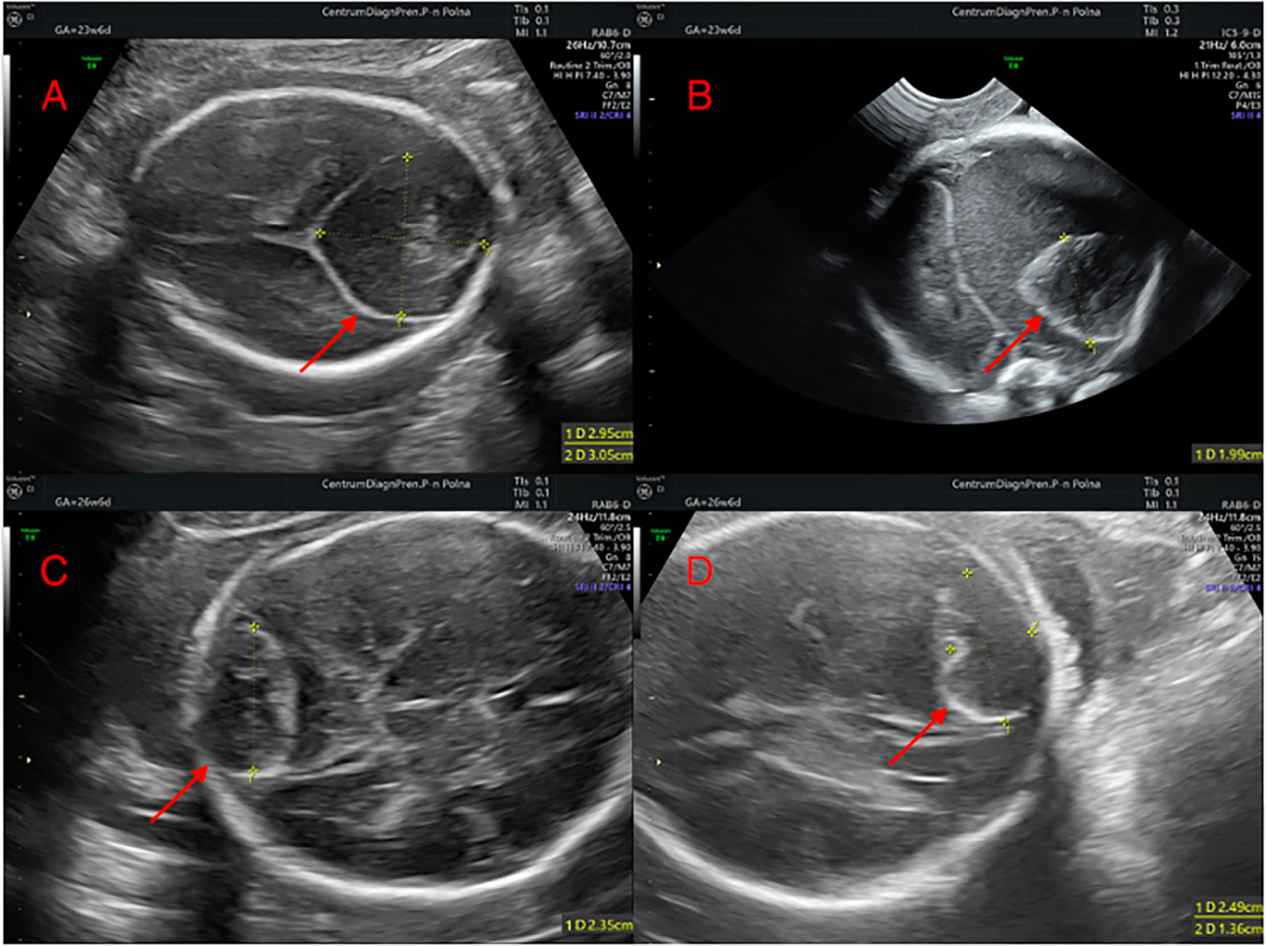
Prenatal ultrasound scans presenting the regression of the posterior fossa cyst: **(A,B)** 23 weeks of gestation; **(C,D)** 26 weeks of gestation.

**Figure 2:**
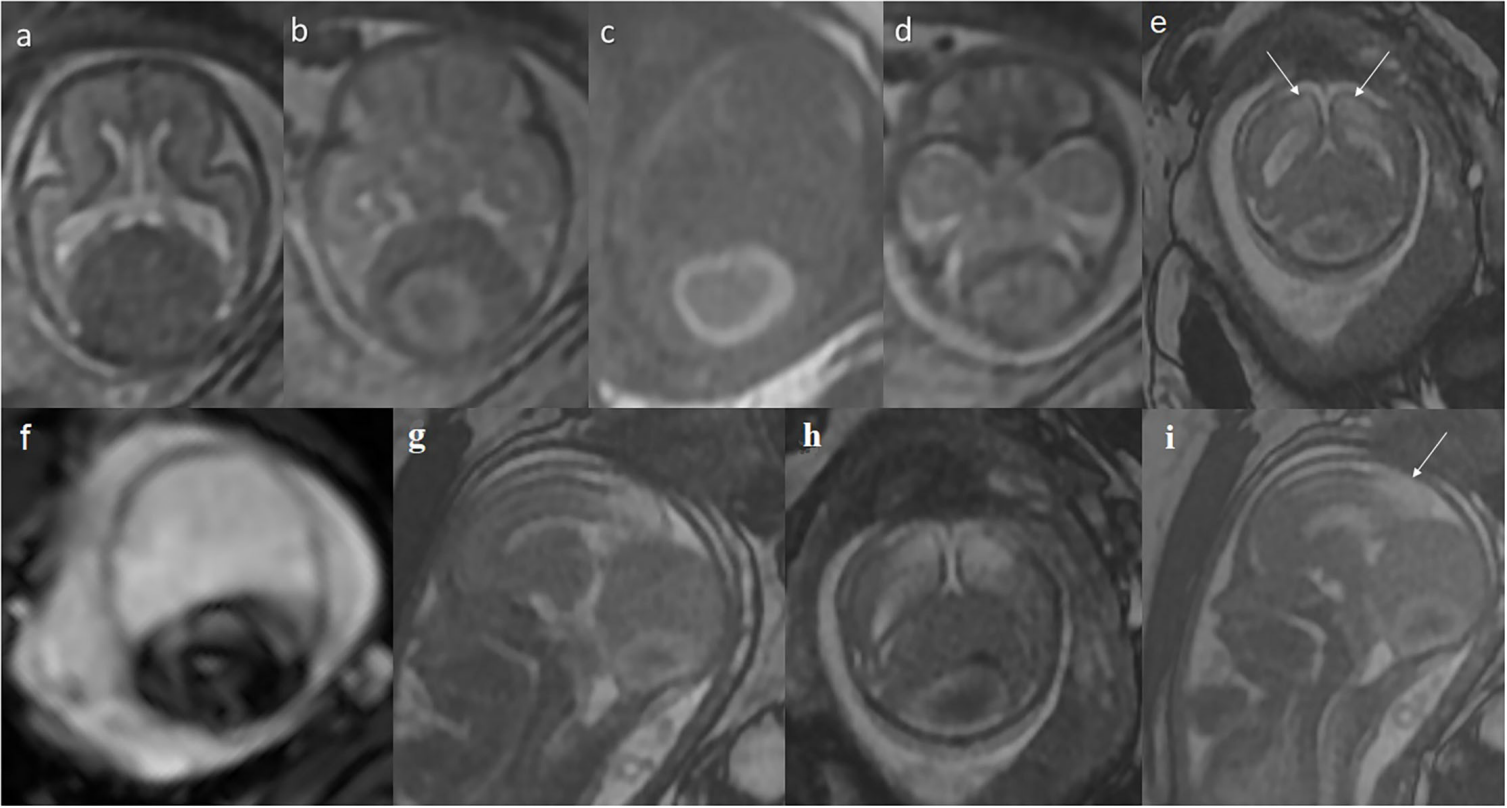
Prenatal MRI at the 23rd gestational week: **(a–f)** axial, **(g,i)** sagittal, and **(h)** coronal plane. Giant widening of the venous sinuses around the sinus confluence. The lesion lifts **(h)** and displaces the cerebral hemispheres apart **(a,b)** and compresses and displaces the posterior cranial fossa structures forward **(d,g)**, with the brain stem pressed against the clivus **(g)**. A thrombus **(c)** containing hemosiderin **(g)** inside. **(e,i)** Abnormal T2-hyperintensity of the posterior-medial parts of the cerebral hemispheres is appreciated (arrows) in FIESTA, indicating ischemia/edema caused by the huge mass of sinus ectasia.

The girl was diagnosed prenatally with a nodular, cystic-solid lesion in the posterior cranial fossa during routine ultrasound scan at the 22nd gestational week (GW) (Figures 1A,B). The mass raised suspicion of either an atypical teratoma or arachnoid cyst. On fetal magnetic resonance imaging (MRI) performed at the 23rd GW, the mass (38 × 35 × 43 mm), with features of internal bleeding (Figure 2c) and thrombosis (Figure 2f), was described as a giant dural venous sinus ectasia. The lesion pulled the cerebral hemispheres apart (Figure 2a), compressed the structures of the posterior cranial fossa (Figure 2d), and pushed the brain stem to the clivus (Figure 2g) but did not cause widening of the ventricular system (Figure 2). In the subsequent prenatal ultrasound examinations, the dimensions of the lesion gradually decreased (Figures 1C,D).

The girl was born *via* C-section at 40 weeks of gestation in good condition, with Apgar score of 10 at the 1st and 5th minute of life and with a birth weight of 2,920 g. During hospitalization, in the first days of life, the physical examination and laboratory values were normal. The brain ultrasound showed neither bleeding nor other changes in the posterior cranial fossa. The brain echogenicity was normal.

**Figure 3:**
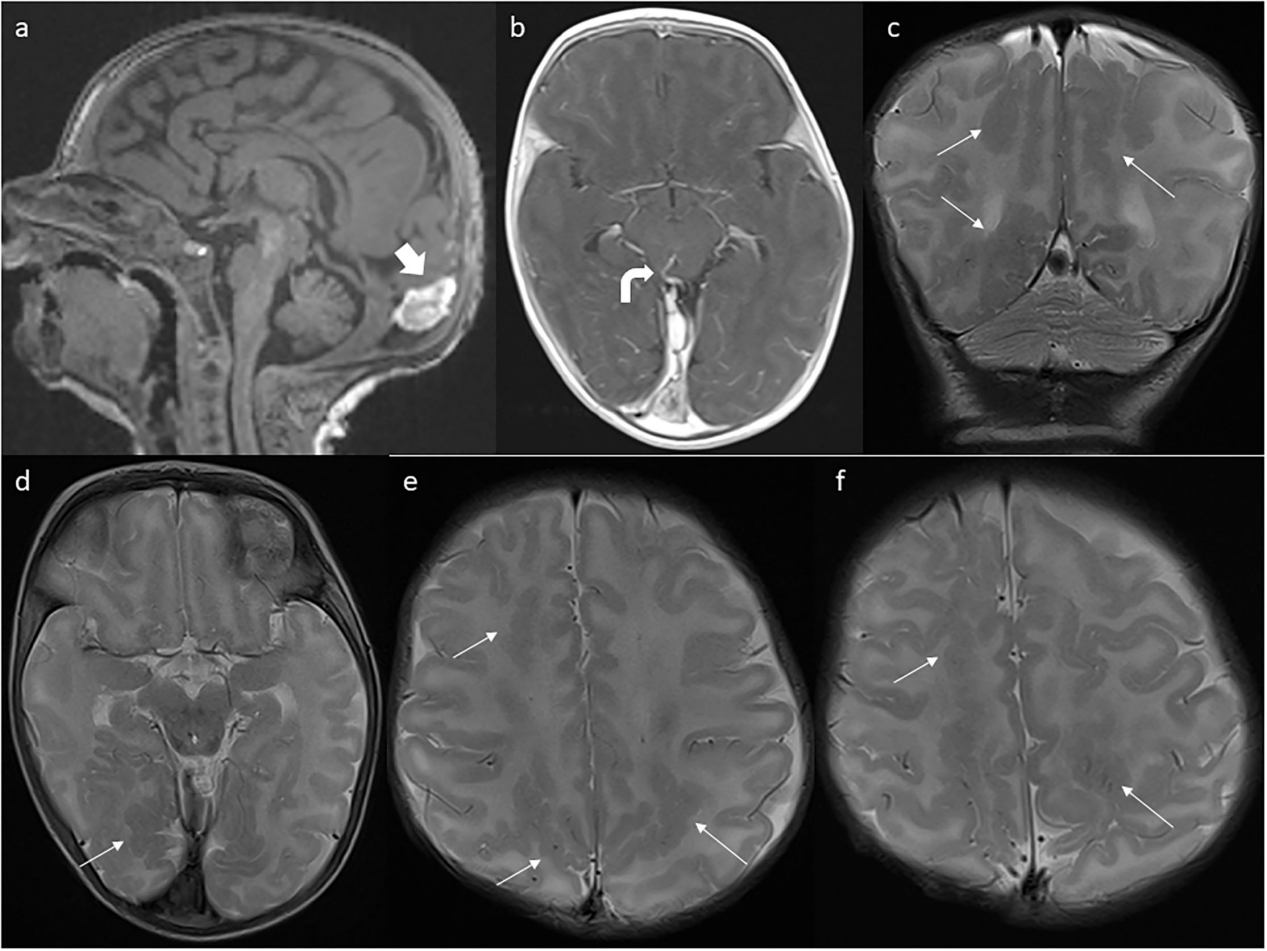
First postnatal MRI scan at the age of 4 weeks. **(a)** Partial regression of dural ectasia (thick arrow). **(b)** One of the abnormal vessels (curved arrow). **(c)** Bilateral parasagittal polymicrogyria (thin arrows) in coronal and **(d–f)** axial planes.

In the 4th week of the girl's life, the brain MRI revealed a torcular pseudomass (20 × 8 × 12 mm) (Figures 3a,b) and deposits of hemosiderin in susceptibility-weighted imaging (SWI) sequence. The most striking finding was diffuse bilateral parasagittal occipital, parietal, and frontal polymicrogyria (Figures 3c–f). Abnormal vessels—probably venous anastomoses—were shown in the dorsal part of the midbrain (Figure 3c) and in the lower part of the left cerebellar hemisphere. On physical examination, hypertonia, tendency to scapular retraction, and epistotonus while in prone position were diagnosed. The patient was referred to the neurologic and neurosurgical outpatient clinics as well as to intensive rehabilitation.

In the 7th week of life, the girl was hospitalized due to a single episode of upper limb ejection and lower limb flexion during a gastrointestinal infection. The EEG during sleep was normal.

**Figure 4:**
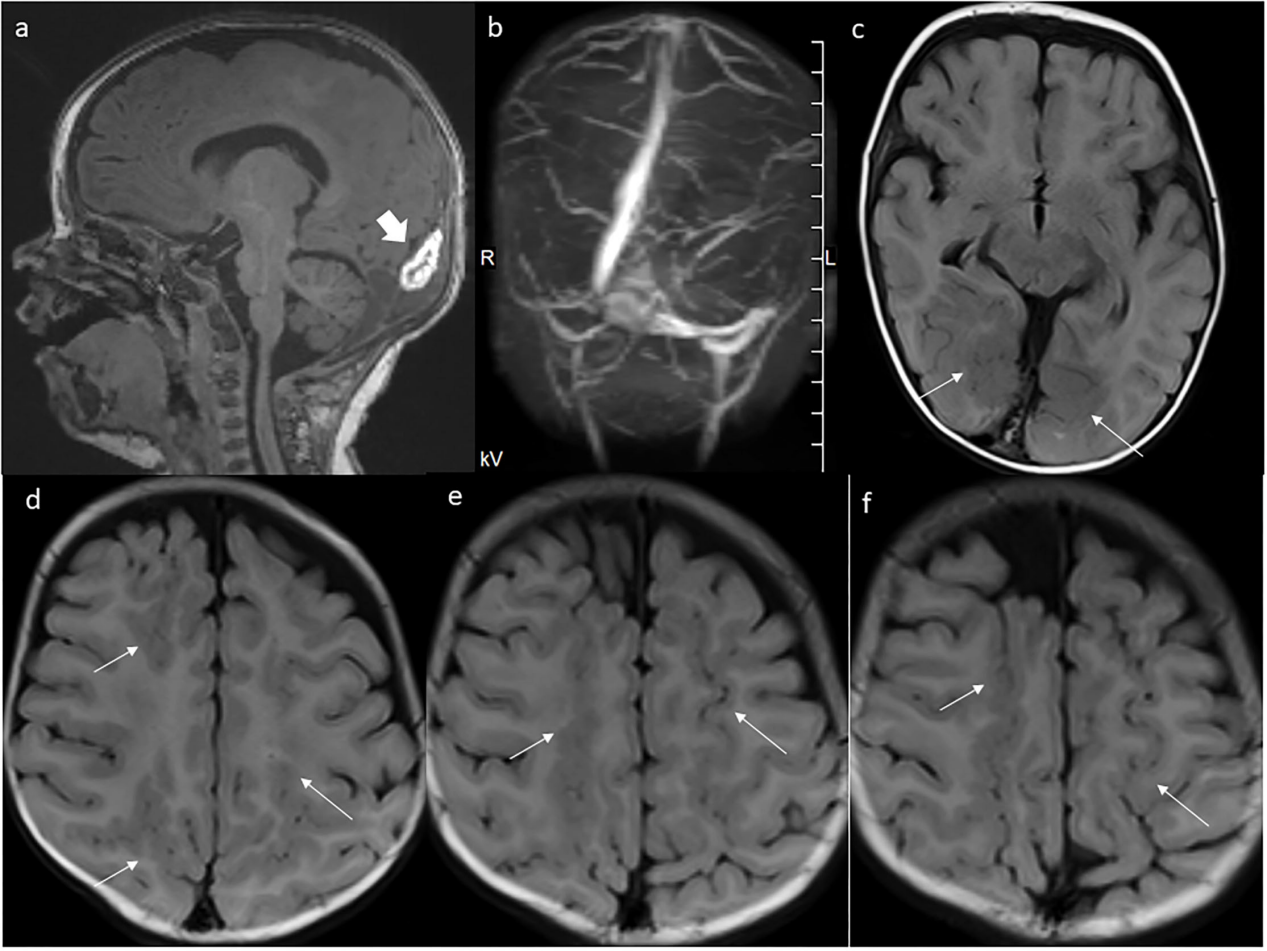
MRI at the age of 5 months. **(a)** Further thinning of the mass. **(b)** Patent dural venous sinuses in MR venography. **(c–f)** The extent of polymicrogyria in the parasagittal parts of the occipital, parietal, and frontal lobes is even better seen now in axial FLAIR images.

In the 5th month of life, a control brain MRI showed a slightly smaller mass within the torcular herophili (23 × 6 × 9 mm; Figure 4a), with smaller hemosiderin deposits in SWI sequence. The MR venography showed the flow in all dural sinuses (Figure 4b). The malformation of cortical development was prominently visualized in FLAIR sequence (Figures 4c–f).

The laboratory diagnostics covered genetic tests for mutations: A1298C and C677T in the MTHFR gene, G1691A in the clotting factor V gene, and G20210A in the prothrombin gene—none of which was found. Moreover, the activity of coagulation factors V, VII, VIII, protein C, and protein S was within normal limits.

At the 12th month of life, despite the malformation of cortical development, the girl's psychomotor development is harmonious, and the head circumference gains are normal.

## Discussion

Dural venous sinus ectasia belongs to a rare group of venous sinus malformations of unknown origin. One of the hypotheses explaining the patomechanism mentions vessel occlusion with thrombotic material causing venous hypertension and arteriovenous anastomoses formation.

Thrombus formation within the cerebral veins can occur in the prenatal and postnatal periods. Possible predisposing risk factors include an immature sinus structure with abnormal endothelium and slow blood flow, maternal coagulation disorders, prematurity, hypoxia, local and systemic inflammatory process, hematological disorders (e.g., deficiency of antithrombin III, protein C, protein S, factor V Leiden mutation, mutation of the prothrombin gene G20210 A, hyperhomocysteinemia, thrombophilia), polycythemia, and trauma (4, 5). Considering the characteristics of the MRI findings in the described case, we assume that the coagulation process occurred *in utero*. Despite the extensive diagnostics performed, no cause was found. Moreover, the underlying genetic etiology of PMG is heterogeneous, and mutations in more than 50 genes have been found. Genetic testing is usually performed when PMG is accompanied by other congenital malformations, dysmorphia (micro- or macrocephaly), developmental delay, or epilepsy and has the highest diagnostic yield when PMG is associated with abnormal head growth. In other cases, whole-exome or whole-genome sequencing might be performed (6). As our patient did not match any of the known phenotypes and early brain ischemia was detected, we waived the genetic testing.

Thrombus developing within the venous sinus malformation may result in brain damage in the ischemic mechanism (5, 7). A potential mechanism explaining the absence of neurological complications may be associated with the anastomoses formation that ensure proper venous blood drainage. In our patient, the abnormal vessels were found on postnatal MRI; however, early brain ischemia must have occurred. The fetal MRI at the 23rd GW shows T2-hyperintensity in the posterior parasagittal parts of both cerebral hemispheres, most likely representing ischemic insult, sufficiently distant in time not to restrict diffusion (Figures 2e,g–i). To the best of our knowledge, we present the first report of polymicrogyria co-existing with giant venous sinus ectasia with thrombus. Taking into consideration all findings in the prenatal imaging (dural abnormality and signs of brain ischemia) and the available knowledge of early ischemia as one of the possible causes of PMG (8), the case of our patient seems to prove the causative association of thrombosed giant dural sinus ectasia with PMG. Moreover, the patient presented with bilateral parasagittal parieto-occipito-frontal polymicrogyria not described before. The most resembling entity found in the literature was bilateral parasagittal parieto-occipital polymicrogyria (9).

The literature covering the topic of giant dural sinus ectasia consists mostly of case reports, in which clinical presentation and outcome differ significantly. The clinical manifestations in the neonatal period include macrocrania, hydrocephalus, seizures, consumptive coagulopathy, spontaneous thrombosis, and cardiac failure (2, 5). None of the above-mentioned manifestations was observed in our patient directly after birth. The prognosis for patients with dural sinus malformations is often poor, with mortality ranging between 38 and 67% (2). However, more recent publications report patients with no or little neurological deficits on follow-up examinations (3, 10). They suggest that, in most cases, dural venous sinus ectasia with thrombosis resolves spontaneously and is not associated with long-term disability (3). Interestingly, the outcome might not be associated with applied treatment and early postnatal symptoms. In the review of 30 cases, the difference between subgroups of neonates and older children is visible: only 26.7% of cases were diagnosed antenatally, of which 75% had a positive outcome, while a generally favorable clinical evolution involved 58.6% of patients (4). Localization of ectasia and thrombosis within the confluence of sinuses does not appear to be related with poor outcome like in the patient described in this report (3).

However, the prognosis in our patient must be considered foremostly in terms of diffuse malformation of cortical development. The wide spectrum of possible clinical manifestations of polymicrogyria ranges from normal individuals with selective impairment of cognitive functions to patients with severe encephalopathies and intractable epilepsy (10). Epilepsy develops in ~80% of patients, in majority within the first 5 years (11). Bilateral involvement, as in our patient, and involvement of more than half of a single hemisphere are poor prognostic factors (9). In the 7th week of life, the reported girl presented with limb ejections and flexions; however, we associate those symptoms with viral gastrointestinal infection at the time as the EEG was normal. The episodes have not been repeated so far, and her neurodevelopment is appropriate for her age.

An initial diagnosis of congenital dural venous sinus ectasia can be made during routine prenatal ultrasound, in which abnormalities within the posterior fossa are described. The differential diagnosis includes tumors, cysts, and Dandy–Walker syndrome (3). Surprisingly, during transfontanelle ultrasound in the first days of life, no abnormalities were found. This suggests that postnatal transfontanelle ultrasound may not always be sufficiently accurate, possibly due to lack of proper insight into the posterior fossa. Based on the available literature, we were able to identify only two cases in which brain ultrasound was mentioned as the imaging method of dural sinus ectasia in the neonatal period (1, 12). A diagnosis of polymicrogyria is possible on fetal ultrasound (13), although it is rarely reached without prenatal MRI. MRI is a method of choice both *in utero* and in the postnatal period for confirmation and for guiding the treatment in both entities described (14).

This report is the first to illustrate the possible causal relationship between giant dural sinus ectasia and polymicrogyria.

## Data Availability Statement

The original contributions presented in the study are included in the article/Supplementary Material, further inquiries can be directed to the corresponding author/s.

## Ethics Statement

Ethical review and approval was not required for the study on human participants in accordance with the local legislation and institutional requirements. Written informed consent to participate in this study was provided by the participants' legal guardian/next of kin. Written informed consent was obtained from the minor(s)' legal guardian/next of kin for the publication of any potentially identifiable images or data included in this article.

## Author Contributions

ZK, PK, and KT-W collected, analyzed, and interpreted the patient's data and had a major contribution in writing the manuscript. BS performed and analyzed the patient's follow-up examination as well as critically revised the manuscript. MP performed, described, and interpreted the prenatal examinations. MS-B contributed to the design of the work and critically revised the manuscript. TS had a major contribution to the conception, drafting, and revising of the manuscript. MB-F retrospectively analyzed and described the patient's neuroimaging results. All authors contributed to the intellectual content of the manuscript, approved its final version, and agreed to be accountable for all aspects of the work.

## Conflict of Interest

The authors declare that the research was conducted in the absence of any commercial or financial relationships that could be construed as a potential conflict of interest.

## Publisher's Note

All claims expressed in this article are solely those of the authors and do not necessarily represent those of their affiliated organizations, or those of the publisher, the editors and the reviewers. Any product that may be evaluated in this article, or claim that may be made by its manufacturer, is not guaranteed or endorsed by the publisher.

## Supplementary Material

Supplementary Figure 1 Scheme presenting the pathophysiology of polymicrogyria and torcular pseudomass development.

Supplementary Figure 2 Timeline.
