# Supplementary figures and images for: Spontaneous Resolution of Congenital Dural Venous Sinus Ectasia Associated With Polymicrogyria—Case Report
Source: Front Pediatr. 2022 Feb 28;10:822551. doi: 10.3389/fped.2022.822551 (PMC8918672; doi:10.3389/fped.2022.822551)

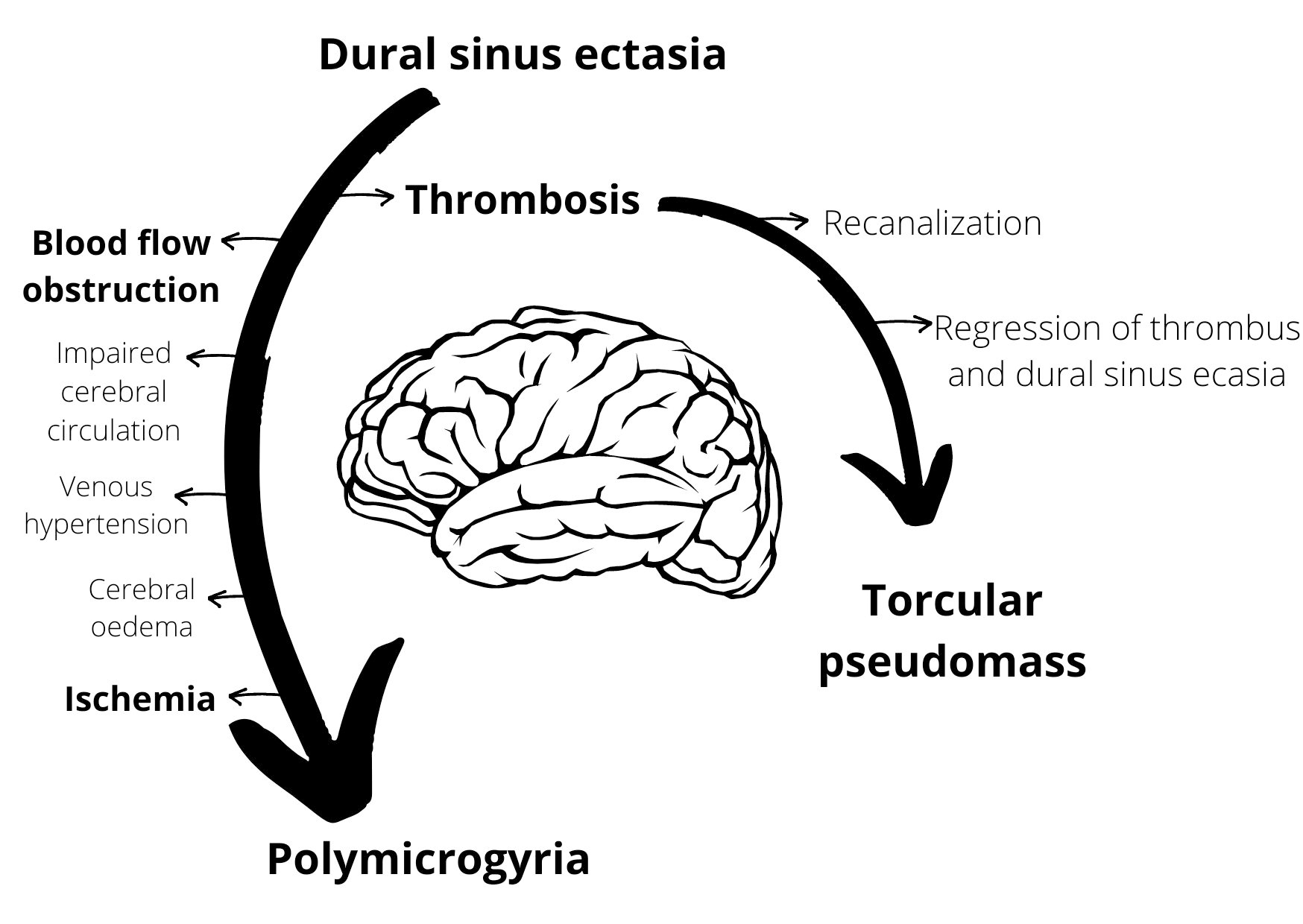

Supplement: Supplementary Figure 1 — Scheme presenting the pathophysiology of polymicrogyria and torcular pseudomass development. [file Image_1.JPEG]

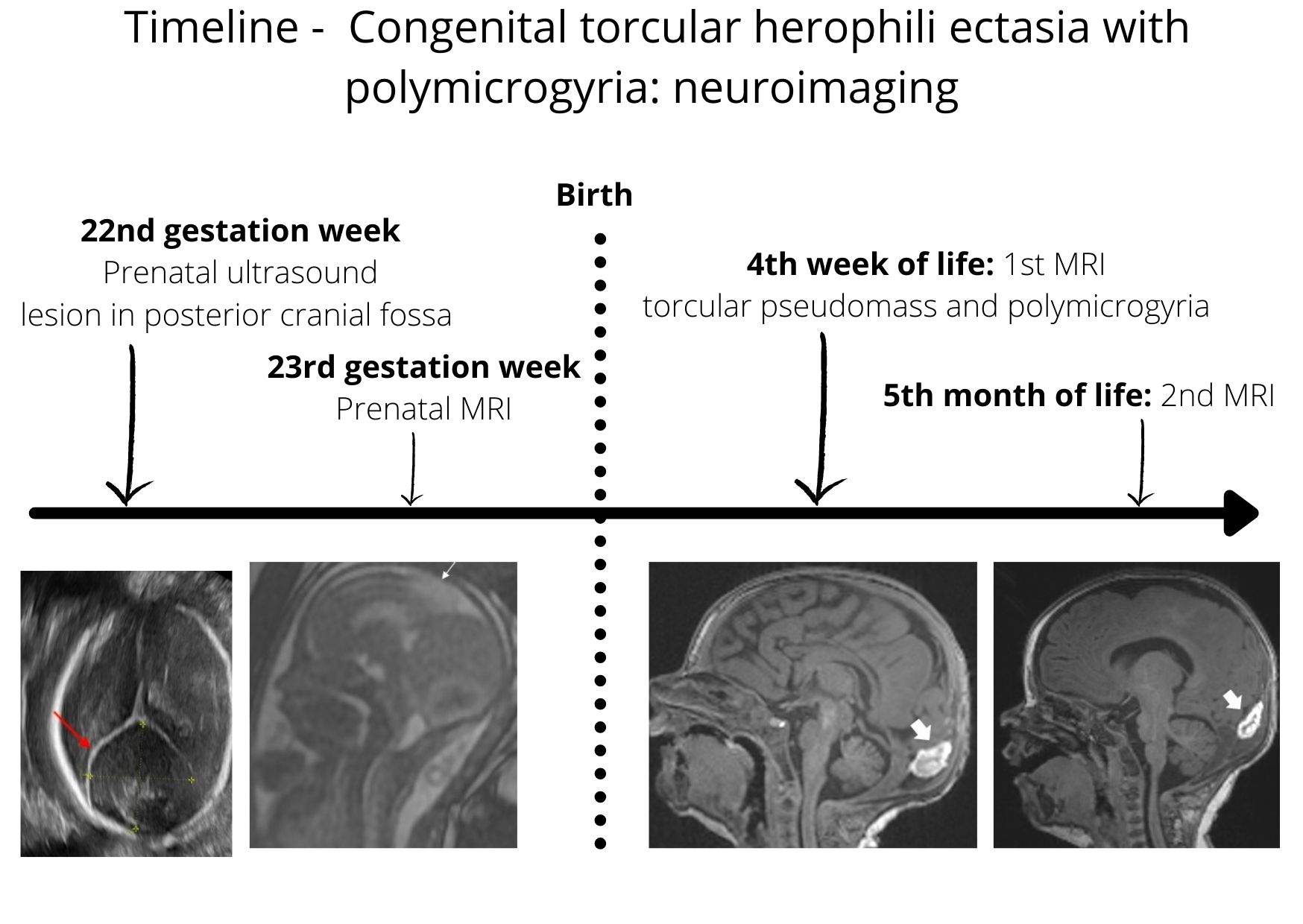

Supplement: Supplementary Figure 2 — Timeline. [file Image_2.JPEG]
